# Gender preference and perinatal depression in Turkey: A cohort study

**DOI:** 10.1371/journal.pone.0174558

**Published:** 2017-03-29

**Authors:** Vesile Senturk Cankorur, Berker Duman, Clare Taylor, Robert Stewart

**Affiliations:** 1 Department of Psychiatry, Ankara University Faculty of Medicine, Ankara, Turkey; 2 King’s College London (Institute of Psychiatry), London, United Kingdom; 3 South London and Maudsley NHS Foundation Trust, London, United Kingdom; Royal Children's Hospital, AUSTRALIA

## Abstract

**Background:**

Child gender preference is important in some cultures and has been found to modify risk for antenatal and postnatal depression. We investigated discrepancies in the child gender preference between participating women and other key family members and the extent to which these predicted perinatal depression.

**Methods:**

In a large cohort study of perinatal depression in urban and rural Turkey, participants had been asked about child gender preferences: their own, and those of their husband, parents, and parents in-law. Of 730 participants recruited in their third trimester (94.6% participation), 578 (79.2%) were reassessed at a mean (SD) 4.1 (3.3) months after childbirth, and 488 (66.8%) were reassessed at 13.7 (2.9) months.

**Results:**

No associations were found between any gender preference reported in the antenatal period and depression at any examination. On the other hand, we found associations of antenatal depression with differences in participant-reported gender preference and that reported for their mother-in-law (OR 1.81, 1.08–3.04). This non-agreement also predicted depression at the 4 month (OR 2.24, 1.24–4.03) and 14 month (OR 2.07, 1.05–4.04) post-natal examinations. These associations with postnatal depression persisted after adjustment for a range of covariates (ORs 3.19 (1.54–6.59) and 3.30 (1.49–7.33) respectively).

**Conclusions:**

Reported disagreement in child gender preferences between a woman and her mother-in-law was a predictor of post-natal depression and may reflect wider family disharmony as an underlying factor.

## Introduction

Postnatal and antenatal depression are common mental disorders. For postnatal depression, a meta-analysis concluded a period prevalence in the three months after birth of 19.2% [1], although there is potentially substantial international variation [2] with particularly high prevalences in Asian countries such as Israel (22.6%), Taiwan (36.6%), Turkey (33,1%; 26,1%) and Vietnam (29.9%) [3–7]. Multiple contributory factors include life stress, marital conflict, maternal self-esteem, and lack of social support [8,9]. Depression or anxiety during pregnancy, past history of psychiatric illness, adverse life events, social support deficits and marital relationship quality have been reported as moderate to strong risk factors for postnatal depression, with obstetric and socioeconomic factors exerting smaller influences [8, 10, 11]. For antenatal depression, life stress, lack of social support, and domestic violence have also been identified as independent risk factors [12].

Research conducted in Western societies has generally found no association between the gender of the child and postnatal depression [11]. However, male gender preference exists in many non-Western cultures [7,13,14,15] and several studies in these settings have found evidence that the birth of a female child when a male child is desired, is associated with postnatal depression [16,17]. Evidence from India [17] and China [18] has also suggested that spousal disappointment with the gender of the baby is significantly associated with postnatal depression in the mother, specifically if the baby is a girl and if the mother has already had a female child. Furthermore, a direct association between male gender preference and antenatal depression has been reported [19], although not by all studies [7, 20]. It has been suggested that the negative reaction of family members towards the birth of a female baby in these contexts may be influential in initiating or exacerbating depression [11, 21].

Perinatal depressive symptoms have been found to be particularly common in Turkey [22] with high prevalence of postnatal depression [23–25]. In general, research in Turkey has tended to confirm risk factors suggested from Western populations such as low income and socio-economic status, previous mental disorders and perceived poor child health. Lack of support from the husband and wider family has also been implicated [5, 6, 25, 26]. It is generally assumed that male gender preference is not as prominent in Turkey as in some of the low and middle income countries in which it has been previously researched; however, it has received little systematic investigation, and may well vary by region and sub-culture. For example, in eastern Turkey a strong male gender preference has been described, and mothers of female babies have been found to have a higher risk of depression [26]. Family preference for a male infant in the previous pregnancy, a female infant in the previous delivery and unwanted pregnancy have also been found to be associated with postnatal depression in western Turkey [27]. As well as those expressed by the woman or her spouse, gender preferences of parents-in-law might also have an effect, because these familial relationships remain societally important; however, these have received little investigation. Furthermore, were gender preferences to exert an influence on maternal mental health, it may be less important whether the gender of the offspring concords with preferences and more important whether there are disagreements in the family unit about such preferences; again, the latter has received little or no investigation but may be particularly salient in cultures where extended family members plays key roles in the perinatal period, whether or not they are co-resident. Using data from a prospective study of perinatal depression from the third trimester onwards, we investigated associations with reported gender preferences and with self-perceived discrepancies in the child gender preference between participating women and other key family members (i.e. the husband, parents and parents-in-law).

## Methods

### Study design, setting and sample

The source cohort study was carried out in Ankara, central Turkey. The principle objective of this original study was to investigate factors associated with antenatal and postnatal depression in Turkish women: in particular focusing on social support from their husband, mother and mother-in-law, gender preferences and to investigate the role of nuclear and traditional family structures in modifying these associations. The study described here was therefore a secondary analysis of pre-existing data. Baseline and first follow-up examinations have been previously described in detail [5,6]. In summary, baseline samples were drawn from 20 urban and semi-rural antenatal clinics, where all women attending routine third trimester antenatal examinations were approached (December 2007 to August 2008). Exclusion criteria were as follows: aged younger than 18 years, illiteracy, and significant physical health problems. Although participants were enrolled to this study through the outpatient clinics where primarily healthy pregnant women receive routine checks, women were asked if there were any significant health problems such as diabetes mellitus, hypertension and thyroid dysfunction. After baseline recruitment and interviewing, attempts were made to re-contact and interview previous participants as close as possible to 2, 12 and 18 months after their childbirth. Follow up interviews were held at participants’ homes. Baseline interviews lasted approximately one hour, the first follow-up 30 minutes, the second follow-up 45 minutes, and third follow-up 1 hour.

Considering sample size calculations, the baseline sample assumed a 25% prevalence of depression as defined by the EPDS and 30% maintenance of depression at 2 months post-partum, and a calculation was made that 750 participants would be sufficient to detect a 0.5 SD group difference in mean primary exposure levels (perceived relationship quality as the original primary objective) between maintained and non-maintained groups (at 80% power and alpha 0.05). The data from the baseline, first and second follow-up examinations are presented in this paper.

The study received approval by ethics committees at Ankara University Faculty of Medicine and King’s College London. After complete description of the study to the subjects, written informed consent was obtained at all examinations. Of 730 participants assessed in their third trimester (94.6% participation rate), 578 (79.2%) were reassessed at a mean (SD) 4.1 (3.3) months after childbirth, and 488 (66.8%) at 13.7 (2.9) months. The main reason for loss to follow-up (16.8%) between the first two examinations was migration of families due to local re-allocation of housing around that time and consequent loss of contact; 37 (5.1%) refused on that occasion. Reasons for loss to follow up (12.4%) between 2 and 12 months postpartum were local re-allocation of housing around that time (7.8%) and refusal (4.6%).

### Measurements

#### Socio-demographic information

Information was obtained as baseline on age, years of education, marital status, current physical health, previous mental health difficulties, life stressors, number of children, and family structure. Because almost all (97.8%) participants were married and cohabiting with their husband, this was not considered as a covariate. Quality of individual relationships was measured using the Close Persons Questionnaire (CPQ) as a primary outcome in initial analyses of this cohort, as reported in previously published papers (5, 6). The realionship quality with the husband was also assessed with a single question; however, this was not included in analyses due to insufficient variance (a very high proportion, 83.1%, reporting this as good or very good).

Self-reported general physical health was ascertained in five groups: very good, good, average, poor and very poor. Previous mental health was categorized as a binary variable on the basis of any self-reported previous diagnosis of depression, other psychiatric illness or past mental health problems. Participants were asked about the presence of the following life stressors/events within the last 12 months, and positive responses were summed and scaled [28]: being in debt, hunger from lack of food, recent separation, problems with friends, recent illness/injury, domestic violence, serious illness in a relative, death of a close family member, death of another relative, problems with a job, problems with money, problems with the justice system, any robbery.

#### Depressive symptoms

The Edinburgh Postnatal Depression Scale (EPDS) had been chosen for this study as one of the most widely used screening instruments for perinatal depression internationally [29] and the most commonly used in previous Turkish research. Although it has been principally applied to assess postnatal depression, it has also been used for antenatal depression [30], and has found to have better screening properties than generic instruments such as the Beck Depression Inventory [30], because of the focus on cognitive features of depression and the avoidance of questions on symptoms such as somatic complaints and sleep disturbance which are problematic as diagnostic items in the perinatal period [29]. The maximum score is 30, and a score of 13 or above was used to classify case-level perinatal depressive symptoms as has been most commonly applied in previous Turkish samples [31, 32].

#### Gender preference

Self-reported gender preference of participants, and participant-reported gender preferences of the husband, mother, father, mother in-law, and father in-law were gathered (i.e. women were asked to report their own views concerning the gender preferences of their relatives). These family members had been chosen as the focus for the wider study due to their important role in marriages in Turkish culture [6]. In the first question women were asked ‘which gender do you want your child to be?’ and could answer one of the following: i) very much prefer male, ii) prefer male, iii) no preference, iv) prefer female, or v) very much prefer female. Other questions were phrased, for example, as ‘which gender does your husband want your child to be’ with the same qualifiers, followed by the same again for the mother, father, mother in-law, and father in-law. Questions about gender satisfaction were asked at follow-up examinations. In order to provide sufficiently informative cell sizes for analysis, all answers were recoded into three groups: male preference, female preference or no preference. Participants’ knowledge of the gender of the baby was also asked at the antenatal examination. For all measures, ‘agreement’ in gender preference was also derived, non-agreement being defined as a different preference between the participant and a given relative (e.g. the participant preferring a male child but reporting female gender preference for her husband); if ‘no preference’ was reported either by the participant or for the relative in question then agreement was concluded.

### Statistical analyses

The sample was initially described with respect to covariates and their associations with depression (using odds ratios and chi-squared tests). Antenatal participant-reported gender preferences and disagreements in this were considered as primary exposures, case-level depressive symptoms were the primary outcome, and covariates were entered sequentially into logistic regression models in the following groups: i) Model 1 adjusting for age only; ii) Model 2 adjusting for age, number of children and duration of education; iii) Model 3 adjusting for age, number of children, duration of education, physical health and number or life stressors; iv) Model 4 adjusting for age, number of children, duration of education, physical health and number or life stressors, and self-reported previous emotional problems. These variables were included on the basis of potential associations with disagreement over gender preference and as potential risk factors for depression.

## Results

### Sample characteristics and associations with depression

In terms of the index pregnancy and childbirth, 78.6% of women reported that the pregnancy was planned, and almost all the participants (99.5%) gave birth at health facilities and 63.3% had a natural delivery without instruments. All participants gave birth to a live baby, but two participants gave birth to twins and one baby died shortly after birth; these were not included analyses. Of the 578 remaining women, 210 (36.1%) had a caesarean section. There were equal proportions of male (50.5%) and female (49.5%) babies, and 97.0% of babies were vaccinated by the time of the first follow-up interview. Of those in extended family settings, 76.2% lived with their mother-in-law, 59.5% with their father-in-law and 57.3% with both. The rest of them were living with their mother, father, siblings or other relatives in different combinations.

Comparing baseline characteristics between groups who were assessed and not assessed at the first follow-up examination, there were no significant differences in age, education level, gender preference, family setting, depression, or any social support measure. Specifically, baseline depression prevalence was 32.6% in those followed and 31.5% in those not followed (chi-squared 0.14, df 1, p = 0.61) and gender preference was also not different between the two groups (no preference, male, and female 48.2%, 23.0%, and 28.8% respectively in women followed, 53.0%, 16.1%, and 30.9% respectively in not followed women, chi-squared 3.39, df 2, p = 0.18). Proportions with no, one, or more than two previous children were 48.9%, 34.5% and 16.6% in women followed, compared to 63.6%, 24.5% and 12.0% respectively in women not followed (chi-squared 17.04, df 2, p = 0.002). The same pattern was observed at the second follow-up: namely, depression prevalence was 31.7% in those followed and 35.4% in those not followed (chi-squared 0.99, df 1, p = 0.32) and gender preference was also not different between the two groups (no preference, male, and female 52.2%, 17.0%, and 30.8% respectively in women followed, 51.6%, 15.9%, and 32.6% respectively in those not followed, chi-squared 0.31, df 2, p = 0.86). Proportions with no, one, or more than two previous children were 47.9%, 34.8% and 17.3% in women followed, compared to 60.8%, 27.0% and 12.2% respectively in women not followed (chi-squared 17.04, df 2, p = 0.002).

Unadjusted associations of covariates with antenatal depression are summarized in Table 1. Depression was associated with higher numbers of previous children, worse reported general health, recent life events/stressors, and self-reported past history of emotional problems. There were no significant associations with age, education level or family structure. Antenatal depression was not significantly associated with gender preference (chi-squared 2.84, df 2, p = 0.42). Furthermore, depression did not significantly differ between women giving birth to male or female babies at at the first and second follow-up examinations after childbirth (respectively, chi-squared 0.48, df 1, p = 0.49; chi-squared 0.01, df 1, p = 0.94).

**Table 1:** Sample characteristics at recruitment (in third trimester of pregnancy) and associations with contemporaneous depressive symptoms.

Baseline characteristics	Mean (SD) or N (%)	Depression prevalence % (n)	Odds ratio	95% confidence intervals
**Age**	26.1 (5.2)			
<22	207 (29.2)	34.3 (71)	Ref.	Ref.
23–25	165 (23.3)	37.6 (62)	1.15	0.75–1.77
26–29	170 (23.9)	25.9 (44)	0.67	0.43–1.05
>30	167 (23.6)	36.5 (60)	1.10	0.72–1.69
**Number of children**				
0	373 (52.4)	31.6 (117)	Ref.	Ref.
1	228 (32.0)	30.3 (69)	0.94	0.66–1.34
≥2	111 (15.6)	46.8 (51)	1.91	1.24–2.93
**Duration of education (years)**	8.4 (3.7)			
≤5	232 (33.3)	33.2 (77)	Ref.	Ref.
6–8	142 (20.4)	33.8 (47)	1.03	0.66–1.60
9–11	239 (34.4)	34.7 (82)	1.07	0.73–1.57
≥12	82 (11.8)	28.0 (22)	0.79	0.45–1.37
**Self-reported physical health**				
Very good	128 (18.0)	30.5 (39)	Ref.	Ref.
Good	442 (62.3)	30.0 (132)	0.98	0.64–1.51
Average or below	140 (19.7)	47.9 (67)	2.09	1.27–3.46
**Life events**				
0	378 (53.1)	25.1 (94)	Ref.	Ref.
1	210 (29.5)	39.5 (82)	1.95	1.36–2.79
2+	124 (17.4)	49.2 (61)	2.88	1.89–4.40
**Any prev. mental health problems**				
No	297 (42.6)	17.5 (52)	Ref.	Ref.
Yes	400 (57.4)	45.8 (183)	3.97	2.78–5.68

### Gender preference / satisfaction and depression

Baseline child gender preferences of the participating women, and those reported for the husband, parents and parents-in-law are displayed in Table 2. No gender preference was reported by 52.2% of women for themselves and their reports of no preference for close relatives ranged from 46.3% for their husband to 75.3% for their fathers. Male gender preference was reported by 16.5% of women for themselves, whereas 34.3% reported male gender preference by their husband. Gender preference was not associated with antenatal depression (chi-squared 2.84, df 2, p = 0.24) or with postnatal depression at follow-up 1 (chi-squared 3.39, df 2, p = 0.18) or follow-up 2 (chi-squared 0.84, df 2, p = 0.66) examinations. No significant associations emerged in women with female babies where there was a male gender preference or vice versa (data not shown).

**Table 2:** Reported child gender preferences/satisfaction for participants, those reported for close relatives, and non-agreements.

	Participant N (%)	Husband N (%)	Mother N (%)	Father N (%)	Mother-in-law N (%)	Father in law N (%)
Baseline preference						
No pref.	379 (52.2)	336 (46.3)	451 (64.4)	508 (75.3)	346 (50.3)	435 (67.2)
Male	120 (16.5)	249 (34.3)	152 (21.4)	113 (16.7)	228 (33.1)	164 (25.3)
Female	227 (31.3)	141 (19.4)	108 (15.2)	54 (8.0)	114 (16.6)	48 (7.4)
Gender satisfaction follow-up 1 Yes/No	563/16 (97.2)	558/19 (96.7)	547/16 (97.2)	535/9 (98.3)	506/39 (92.8)	489/24 (95.3)
Gender satisfaction follow-up 2 Yes/No	472/8 (98.3)	470/8 (98.3)	469/8 (98.3)	458/4 (99.1)	446/20 (95.2)	436/12 (97.3)

Considering measures taken at the first and second follow-up examinations after childbirth, there were generally very high levels of gender satisfaction reported for all family members with only that for the mother-in-law at the first follow-up falling below 95%. Associations between the mother-in-law dissatisfaction and maternal depression at first follow-up examinations were significant (chi-squared 12.71, df 1, p<0.01) whereas the analyses could not perform for the second follow-up examination due to small numbers.

#### Gender preference non-agreement at baseline and its association with antenatal and postnatal depressive symptoms

Considering non-agreements at the baseline assessment between participants’ gender preference and those perceived for their family members, displayed in Table 3, the highest likelihood of non-agreement was between a participant and her husband (12.5%) and followed by mother in-law (10.9%). Associations of non-agreements in child gender preference at baseline with depressive symptoms at baseline and at the two follow-up points are also summarised in Table 3. The only statistically significant associations found were between between non-agreement with the mother-in-law at baseline and depression at all three examinations. In terms of moderation analyses, associations between non-agreement with the mother-in-law and maternal depression were not significant between first time mothers (chi-squared 2.89, df 1, p = 0.09) and those who already have a child (chi-squared 1.99, df 1, p = 0.16) in antenatal examinations. On the other hand, these associations were different between first time mothers (chi-squared 7.61, df 1, p = 0.01) and those who already have a child (chi-squared 1.11, df 1, p = 0.29) at the first follow-up examinations. Furthermore, these associations were different for those living in traditional families (chi-squared 11.12, df 1, p<0.01) compared to nuclear families (chi-squared 0.77, df 1, p = 0.38) at the first follow-up assessments, whereas these associations were not different for those living in traditional families (chi-squared 1.99, df 1, p = 0.16) compared to nuclear families (chi-squared 2.24, df 1, p = 0.13) at antenatal assessments.

**Table 3:** Non-agreement in child gender preference at baseline (third trimester) and associations with depressive symptoms at baseline and each follow-up.

Non-agreement in gender preference between the participant and the stated relative	Number	Depression prevalence (%)		
		Baseline examination (third trimester)	Follow-up 1 examination (4 months post-partum)	Follow-up 2 examination (14 months post-partum)
**Husband**				
No	635	32.6	24.9	25.6
Yes	91	40.4	30.2	29.4
**Mother**				
No	611	31.9	24.6	24.9
Yes	49	33.3	34.3	40.0
**Father**				
No	537	31.5	24.8	25.6
Yes	38	31.6	35.7	30.4
**Mother in law**				
No	629	30.9	23.5	23.7
Yes	77	42.7	40.7	39.0
**Father in law**				
No	633	31.2	23.0	23.4
Yes	51	34.0	29.7	26.7

Prospective analyses are summarised in Table 4. Child gender preference non-agreement with the mother in-law at baseline was associated with postnatal depression at the first follow-up examination and at the second follow-up. No associations were found for non-agreements with any other relative. Adjusted associations for non-agreements with the husband and mother in law at all three examinations are displayed in Table 4. In the final, fully adjusted model, postnatal depression at the first and second follow-up examinations remained significantly associated with gender preference non-agreement with the mother in-law, but there were no associations for non-agreement with the husband in fully adjusted models.

**Table 4:** Adjusted associations of baseline non-agreement with husband and mother in law and depressive symptoms at baseline, follow-up 1, follow-up 2.

	Unadjusted OR (95% CI)	Model 1 OR (95% CI)	Model 2 OR (95% CI)	Model 3 OR (95% CI)	Model 4 OR (95% CI)
**Non-agreement with the husband and baseline depression**	1.45	**1.65**	1.54	1.55	1.46
	(0.91–2.28)	**(1.03–2.64)**	(0.94–2.51)	(0.91 -.2.65)	(0.84–2.56)
	(n = 708)	(n = 694)	(n = 673)	(n = 604)	(n = 589)
**Non-agreement with the mother-in-law and baseline depression**	**1.67**	**1.94**	**1.85**	1.73	1.85
	**(1.02–2.73)**	**(1.16–3.22)**	**(1.09–3.16)**	(0.94–3.17)	(0.97–3.53)
	(n = 616)	(n = 603)	(n = 586)	(n = 552)	(n = 509)
**Non-agreement with the husband and follow-up 1 depression**	1.29	1.43	1.41	1.30	1.28
	(0.73–2.31)	(0.79–2.59)	(0.76–2.60)	(0.67–2.52)	(0.66–2.50)
	(n = 540)	(n = 531)	(n = 519)	(n = 467)	(n = 454)
**Non-agreement with the mother-in-law and follow-up 1 depression**	**2.24**	**2.50**	**2.55**	**2.82**	**3.19**
	**(1.24–4.03)**	**(1.36–4.61)**	**(1.35–4.79)**	**(1.40–5.71)**	**(1.54–6.59)**
	(n = 475)	(n = 466)	(n = 455)	(n = 406)	(n = 395)
**Non-agreement with the husband and follow-up 2 depression**	1.21	1.28	1.21	1.33	1.30
	(0.64–2.31)	(0.66–2.46)	(0.62–2.39)	(0.65–2.70)	(0.64–2.66)
	(n = 442)	(n = 435)	(n = 422)	(n = 382)	(n = 373)
**Non-agreement with the mother in-law and follow-up 2 depression**	**2.07**	**2.19**	**2.12**	**3.11**	**3.30**
	**(1.05–4.04**)	**(1.10–4.34)**	**(1.04–4.32)**	**(1.43–6.77)**	**(1.49–7.33)**
	(n = 395)	(n = 388)	(n = 376)	(n = 339)	(n = 331)

p≤0.05 (bold); baseline = third trimester; follow-up 1 = mean 4 months post-partum; follow-up 2 = mean 14 months post-partum

Model 1. Adjusted for age

Model 2. Adjusted for 1 and number of children, duration of education

Model 3. Adjusted for 2 and physical health, number of life events/stressors

Model 4. Adjusted for 3 and previous emotional problem

## Discussion

Child gender preference is an important issue in some cultures [5,7] and it has been reported as a risk modifier for antenatal and postnatal depression in studies in some countries [7,17], although is generally under-researched. Findings to date have tended to come from societies where there are marked contrasts between the implications of a male or female child, and the exposure has received little or no investigation in Western settings where preferences, if present, are assumed not to have meaningful impact. We took advantage of a large Turkish cohort to investigate the issue in a potentially ‘intermediate’ setting, where there is recognised to be some level of gender preference but probably not as extreme as in other cultures. To our knowledge, ours is the first study to assess the association prospectively or across such a range of family members, although it has been investigated previously in cross-sectional studies [27].

Child gender preference is not a unitary construct, but potentially reflects a spectrum of differing views from relevant family members. This has not been well captured in previous research. Therefore, we took advantage of a cohort study in which mothers were asked not only about their own preference but also about the perceived preferences of five other close family members, and we not only analysed specific reported preferences as exposures but also reported differences in preferences. We acknowledge that all these preferences were those perceived by the participant, and not necessarily those actually held by family members; however, in relation to participants’ mental health, the perception is likely to be more important than the actuality. We were additionally able to describe reported gender satisfaction after the birth, although there was insufficient variance in this for analysis. In our sample, an absence of child gender preference was relatively common, reported by just over half of women for themselves. Mothers’ reports of no preference by a close relative ranged from just under half for their husband to three quarters for their fathers. Male gender preference was reported by 17% of women for themselves, whereas twice this proportion reported male gender preference by their husband. When questions were asked about satisfaction with the gender of the child in the postnatal follow up exampinations, there were generally very high levels of gender satisfaction reported for all family members. While it is possible that participants were reluctant to report gender preference, we feel that this is unlikely, given that interviewers were drawn from similar backgrounds and interviews conducted in private environments. Additionally, there is no significant social stigma associated with gender preference which would give rise to under-reporting.

Despite giving birth to girls being a risk factor in some low and middle income countries [17, 27], gender preference was not significantly associated with antenatal or postnatal depression. Furthermore, giving birth to male or female babies was not associated with depression at any examination after childbirth. The lack of an association between gender preference and depressive symptoms was unexpected considering that societal preference for sons is reported in Turkey where the the family's preference for a male infant in the previous pregnancy and female infant in the previous delivery were associated with postnatal depression (respectively, OR 3.10, 1.57–6.12 and OR 2.18, 1.09–4.37) [27]. A possible explanation for no association being found between gender preference and perinatal depression in our sample might be the lack of gender preference found.

Associations between the child gender preference of relatives and maternal depression have been noted in India and China [17,33]. Specifically, Xie et al. [33] suggested that the association between the gender of the baby and postnatal depression found in their study was not due to the baby’s gender itself, but rather the social context and reactions of relatives to the baby. In a recent study, preference for a male child, previously suggested to be influential in Vietnam, was not linked to depressive symptoms; instead, poor family relationships and negative reactions to the infant were associated with lowered maternal wellbeing [7]. Intense levels of postpartum support from relatives have been described as a ‘double edged sword’ [18], and being in a close relationship with disapproving or emotionally cold relatives during the postnatal period may act as a substantial stressor. In the aforementioned Vietnamese study, it was reported that a relative (particularly the mother-in-law) having negative reactions to the infant, was associated with decreased wellbeing of women in the postnatal period [7].

Despite no associations being found for child gender or gender preference with perinatal depression, we did find some associations between depressive symptoms and participant-reported non-agreements in child gender preference with their mother in-law. Of note, all adjusted odds ratios in Table 4 are above one, suggesting that some level of disagreement may represent a risk factor for depression, although disagreements with the mother in law showed the strongest and most consistent relationships. Furthermore, these associations were modified by having a child or not and living in traditional or nuclear family settings after giving birth. Gender preference non-agreement with the mother-in-law predicted postnatal depression at the 4 month examination, independent of a range of covariates, whereas gender preference conflict with the husband did not predict postnatal depression. The more demonstrable associations concerning relationships with the mother-in-law are consistent with the importance of this figure in women’s lives in this culture and are also consistent with our previously published findings on relationship quality as a risk factor for depression [5,6]. Lower emotional support from the mother-in-law was associated with incidence of postnatal depression [6]. Support from family members has been found to be an important buffer against depression in women from other low and middle-income settings [5,6] and gender child disagreement with mother-in-law may effect support from mother-in-law. Another explanation might be that the perceived relationship with the mother-in-law is more strongly linked with the quality of a woman’s marriage. Gender preference conflicts with the mother, father and father in-law were not associated with antenatal depression and did not predict postnatal depression. The lack of association with these disagreements, despite appreciable prevalence, might indicate relatively low perceived family pressure on women and might account for the overall lack of association between child gender, family gender preference and maternal depression.

Strengths of this study include its prospective design, the particular features of the setting and society for the purposes of these research questions, the large and heterogeneous sample, the standardised assessment instruments which have been well-validated in a variety of international settings, and a comprehensive range of covariates. As a secondary analysis of data primarily collected for investigating social support and depression outcome, the sample was not specifically assembled for the objectives described here; however, post-hoc power calculations based on agreement or not with the spouse and mother-in-law as exposures, indicated 80% power to detect a 60% risk ratio for depression at first follow-up as an outcome (at alpha 0.05). The cohort was therefore felt to be of a sufficient size to detect meaningful effects, although did not permit stratification by the child’s gender and birth order. Response rates were relatively high and we believe that the findings should generalise to the source populations. The Edinburgh Postnatal Depression Scale is widely used in international research; however, it should be borne in mind that it is a screening instrument, measuring number of depressive symptoms, and does not seek to define specific depression syndromes. There are also potentially complex multidirectional relationships between perinatal depression and gender inequality, life events, previous mental health, physical health, social support, and family structures. Further research using designs that can determine the direction and strength of these relationships is recommended. Finally, it is important to bear in mind the fact that all family measures were self-reported by the participants and it is possible that depressive symptoms may have exaggerated the way relationships, and the attitudes of other family members were appraised and reported.

There has been considerable concern around changes in family structures over the last 100 years and their impact on mental health. Turkey, in common with other Middle Eastern countries has been particularly affected, although such cultural changes have been occurring over a relatively longer period. To our knowledge, ours is the first prospective study of this nature from a Middle Eastern setting and we believe that our findings provide a template for further research both in Turkey and elsewhere. Because these findings are novel and requiring replication, clinical implications need to be considered with caution. However, it would be reasonable to propose that discussions around attitudes to the gender of the infant, in societies and cultures where this is salient, should not only encompass the mother’s feelings but also identify those preferences reported for other family members, in addition to corroborating such information from the family members themselves. It is possible that disagreements around gender preferences may reflect more important underlying strains in family relationships; however, this may still represent a way in which such strains might be identified and addressed through the therapeutic relationship. Our study further supports the salience of the extended family environment in perinatal depression, and potentially in women’s mental health more widely, and at least raises the possibility of a focus for preventative intervention.

## Conclusion

In this cohort of women from urban and rural communities in Turkey followed from the third trimester of pregnancy through to the post-natal period, no association was found between gender preference reported by the woman, or that reported for any close relative, in the antenatal period and depression at any examination. On the other hand, we did find associations of antenatal depression with differences in gender preference reported by participants and those reported for their mother-in-law, which also predicted depression at 4- and 14-month post-natal examinations. These associations persisted after adjustment for a range of covariates. These reported disagreements in gender preference might reflect wider family disharmony and would be worth investigating further in cultures where extended family relationships are highly salient for women following childbirth. However, it should be borne in mind that numbers of multiparous women were not sufficient for subgroup analyses, taking into account child and sibling genders. These might well modify associations, but would need investigating in more specific cohorts.

## Supporting information

S1 File PLOSdata. (SAV)

S2 File PLOS.statistics. (DOC)
