# Supplementary material for: Gender preference and perinatal depression in Turkey: A cohort study
Source: PLoS One. 2017 Mar 29;12(3):e0174558. doi: 10.1371/journal.pone.0174558 (PMC5371330; doi:10.1371/journal.pone.0174558)
Supplement: S2 File — (DOC) [file pone.0174558.s002.doc]

| **Statistics** | | | | | | |
| --- | --- | --- | --- | --- | --- | --- |
|  | | {{{{genpremlf1}}}} q52 gender prefernce of mother in law | q48 gender prefernce of mother in law | EPDSF2TOTCAT | genderdifferpregmotl | EPDSF1CAT |
| N | Valid | 545 | 466 | 466 | 688 | 578 |
|  | Missing | 231 | 310 | 310 | 88 | 198 |

| **{{{{genpremlf1}}}} q52 gender prefernce of mother in law** | | | | | |
| --- | --- | --- | --- | --- | --- |
|  | | Frequency | Percent | Valid Percent | Cumulative Percent |
| Valid | 1 | 506 | 65.2 | 92.8 | 92.8 |
|  | 2 | 39 | 5.0 | 7.2 | 100.0 |
|  | Total | 545 | 70.2 | 100.0 |  |
| Missing | System | 231 | 29.8 |  |  |
| Total | | 776 | 100.0 |  |  |

| **q48 gender prefernce of mother in law** | | | | | |
| --- | --- | --- | --- | --- | --- |
|  | | Frequency | Percent | Valid Percent | Cumulative Percent |
| Valid | 1 | 446 | 57.5 | 95.7 | 95.7 |
|  | 2 | 20 | 2.6 | 4.3 | 100.0 |
|  | Total | 466 | 60.1 | 100.0 |  |
| Missing | System | 310 | 39.9 |  |  |
| Total | | 776 | 100.0 |  |  |

| **EPDSF2TOTCAT** | | | | | |
| --- | --- | --- | --- | --- | --- |
|  | | Frequency | Percent | Valid Percent | Cumulative Percent |
| Valid | .00 | 344 | 44.3 | 73.8 | 73.8 |
|  | 1.00 | 122 | 15.7 | 26.2 | 100.0 |
|  | Total | 466 | 60.1 | 100.0 |  |
| Missing | System | 310 | 39.9 |  |  |
| Total | | 776 | 100.0 |  |  |

| **Case Processing Summary** | | | | | | |
| --- | --- | --- | --- | --- | --- | --- |
|  | Cases | | | | | |
|  | Valid | | Missing | | Total | |
|  | N | Percent | N | Percent | N | Percent |
| {{{{genpremlf1}}}} q52 gender prefernce of mother in law * EPDSF1CAT | 542 | 69.8% | 234 | 30.2% | 776 | 100.0% |

| **{{{{genpremlf1}}}} q52 gender prefernce of mother in law * EPDSF1CAT Crosstabulation** | | | | |
| --- | --- | --- | --- | --- |
| Count | | | | |
|  | | EPDSF1CAT | | Total |
|  |  | .00 | 1.00 |  |
| {{{{genpremlf1}}}} q52 gender prefernce of mother in law | 1 | 384 | 120 | 504 |
|  | 2 | 19 | 19 | 38 |
| Total | | 403 | 139 | 542 |

| **Chi-Square Tests** | | | | | |
| --- | --- | --- | --- | --- | --- |
|  | Value | df | Asymp. Sig. (2-sided) | Exact Sig. (2-sided) | Exact Sig. (1-sided) |
| Pearson Chi-Square | 12.711^a^ | 1 | .000 |  |  |
| Continuity Correction^b^ | 11.375 | 1 | .001 |  |  |
| Likelihood Ratio | 11.197 | 1 | .001 |  |  |
| Fisher's Exact Test |  |  |  | .001 | .001 |
| Linear-by-Linear Association | 12.688 | 1 | .000 |  |  |
| N of Valid Cases | 542 |  |  |  |  |
| a. 0 cells (.0%) have expected count less than 5. The minimum expected count is 9.75. | | | | | |
| b. Computed only for a 2x2 table | | | | | |

| **Case Processing Summary** | | | | | | |
| --- | --- | --- | --- | --- | --- | --- |
|  | Cases | | | | | |
|  | Valid | | Missing | | Total | |
|  | N | Percent | N | Percent | N | Percent |
| gendifpregmatlcat * EPDSCAT13 | 616 | 79.4% | 160 | 20.6% | 776 | 100.0% |

| **gendifpregmatlcat * EPDSCAT13 Crosstabulation** | | | | |
| --- | --- | --- | --- | --- |
| Count | | | | |
|  | | EPDSCAT13 | | Total |
|  |  | .00 | 1.00 |  |
| gendifpregmatlcat | .00 | 147 | 49 | 196 |
|  | 1.00 | 78 | 39 | 117 |
|  | 2.00 | 110 | 51 | 161 |
|  | 3.00 | 43 | 32 | 75 |
|  | 4.00 | 39 | 28 | 67 |
| Total | | 417 | 199 | 616 |

| **Chi-Square Tests** | | | |
| --- | --- | --- | --- |
|  | Value | df | Asymp. Sig. (2-sided) |
| Pearson Chi-Square | 11.307^a^ | 4 | .023 |
| Likelihood Ratio | 11.251 | 4 | .024 |
| Linear-by-Linear Association | 9.547 | 1 | .002 |
| N of Valid Cases | 616 |  |  |
| a. 0 cells (.0%) have expected count less than 5. The minimum expected count is 21.64. | | | |
